# Design of Electrochemically Effective Double-Layered Cation Exchange Membranes for Saline Water Electrolysis

**DOI:** 10.3390/polym12092114

**Published:** 2020-09-17

**Authors:** In Kee Park, Dong-Hoon Lee, Chang Hyun Lee

**Affiliations:** 1 Energy Engineering Department, Dankook University, Cheonan 31116, Korea; inkee0149@gmail.com; 2 Department of Chemical Engineering, College of Engineering, Wonkwang University, Iksan, Jeonbuk 54538, Korea; merits98@wku.ac.kr

**Keywords:** saline water electrolysis, double-layered cation exchange membrane, UV irradiation, OH^−^ ion permeability, caustic current efficiency

## Abstract

Saline water electrolysis (SWE) is an electrochemical process to simultaneously produce hydrogen (H_2_), chlorine (Cl_2_), and sodium hydroxide (NaOH) with high purity levels (e.g., 99.999%) by applying electric power to saline water. The state-of-the art SWE membrane, Flemion^®^, has excellent chemical resistance to harsh SWE conditions, but still needs to lower its energy consumption by reducing its ohmic resistance to Na^+^ ion transport. Meanwhile, most of cation exchange membranes (CEMs) have been suffering from chemical degradation under the alkaline conditions, owing to their single layer matrices composed of sulfonic acid moieties, though they show fast Na^+^ ion transport behavior. Here double-layered SWE membranes were prepared on the basis of design strategies composed of the incorporation of a chemically stable carboxylic acid layer (C layer) via UV irradiation onto one surface of perfluorinated Nafion^®^212 membrane chosen as one of commercially available CEMs, and the thickness control of the C layer. The resulting membranes showed excellent SWE performances and improved electrochemical service life, when compared with those of Nafion^®^212 and Flemion^®^, respectively.

## 1. Introduction

Saline water electrolysis (SWE) is an electrochemical conversion process to simultaneously generate highly pure hydrogen (H_2_), chlorine (Cl_2_), and sodium hydroxide (NaOH) by applying electric power to saline water [1,2,3]. Here, saline water is defined as water in which alkali metal or alkali earth metal cations and their counter anions (e.g., Na^+^ and Cl^−^ ions) are dissolved in the concentration range from 3.5 to 25 wt % [4]. SWE has been also used in a wide range of fields including CO_2_ mineralization [4,5] and ballast water disinfection [6,7]. In spite of its industrial importance, SWE consumes a large amount of energy to be known as a representative energy-intensive technology. The high energy consumption arises from the big differences between theoretical cell voltage (i.e., 2.2 V) and practical cell voltage levels (e.g., 3.8–4.0 V) defined in terms of overpotentials.

Up to now, there have been continuous approaches to lower SWE energy consumption by reducing the overpotentials. They include the use of SWE cells employing fuel cell configuration [8], the zero-gap method reducing interfacial resistance between SWE cell components [9], the porous electrodes with easy H_2_ and Cl_2_ generation and emission [10], and SWE membranes with low ohmic resistance [5,11]. Among them, it is very effective to reduce the SWE membrane resistance, because Na^+^ ions, targeted mobile ions in SWE, are more slowly transported through SWE membranes than through other medium including anolyte and catholyte. Thus, the desirable membrane should be designed in the form of cation exchange membranes (CEMs) to have high Na^+^ ion conductivity. The SWE membrane must also be able to prevent the diffusion of OH^−^ ions as much as possible. Otherwise, the OH^−^ ions permeated from cathode to anode through SWE membranes not only hinder NaOH production, but also reduce the corresponding energy efficiency called as caustic current efficiency. From the material point of view, permeated OH^−^ ions induce severe chemical degradation to SWE membranes, particularly with single layer matrices made up of sulfonic acid moieties. For this, the desirable SWE membranes should have excellent resistance to alkaline attacks under strong base atmospheres. The state-of-the art SWE membrane to satisfy these tough requirements is Flemion^®^ (AGC Chemicals Company, Japan). Flemion^®^ is prepared in the form of double-layered CEM composed of thick perfluorinated sulfonic acid (PFSA) ionomer layer (S layer) and thin perfluorinated carboxylic acid (PFCA) ionomer layer (C-layer). Even Flemion^®^ needs to be enhanced further to ensure high electrochemical SWE performances including energy efficiency.

In this study, SWE membrane design strategies were demonstrated to develop alterative SWE membranes to Flemion^®^. The strategies are based on the incorporation of a chemically robust C layer via free radical polymerization under UV irradiation on one surface of Nafion^®^212 membrane, one of commercially available CEMs, and the thickness control of the C layer, to reduce the diffusion of OH^−^ ions, and to find out an appropriate C layer thickness considering both SWE electrochemical performances and service life, respectively. The major purpose of this study is to confirm whether the design strategies are applicable. Another objective is to disclose the effects of the C layer thickness on fundamental and electrochemical characteristics of the resulting double-layered SWE membranes. The final goal is to monitor electrochemical service life of the double-layered SWE membrane with the optimum C layer thickness.

## 2. Materials and Methods

### 2.1. Materials

Acrylic acid (AA, purity = 99.0%) and divinyl benzene (DVB, purity = 99.0%) were purchased from Sigma-Aldrich (Verona, WI, USA). Benzoin isobutyl ether (BIE, purity = 94.0%) and α,α’-azobis(isobutyronitrile) (AIBN, purity = 98.0%) as free radical initiators were purchased from TCI Chemicals (Tokyo, Japan) and Sigma-Aldrich, respectively. All the chemicals except AA were used as received without any further purification. AA was purified by removing hydroquinone monomethyl ether used as an inhibitor via the distillation at 55 °C under the pressure of 6 torr. Nafion^®^212 with the equivalent weight of 1100 meq g^−1^ was also purchased from Chemours (Wilmington, DE, USA) and used as both a reference and S layer for double-layered SWE membrane formation.

### 2.2. Double-Layered SWE Membrane Formation

**Figure 1:**
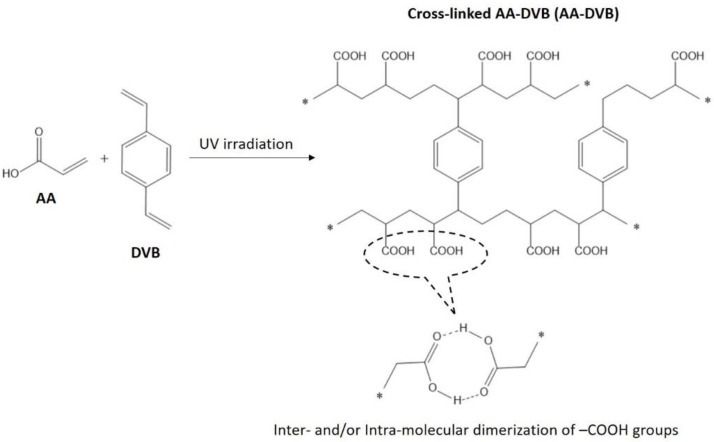
Chemical structure of AA-DVB polymeric network used as the C layer.

The C layer was synthesized via free radical polymerization under ultraviolet (UV) irradiation [12,13] as shown in Figure 1. For this, the mixture of AA, DVB, BIE, and AIBN with the molar ratio of 2 (7.28 g), 1 (6.58 g), 0.1 (0.84 g), and 0.1 (1.43 g) was stirred in a three-neck flask containing a reflux condenser under nitrogen atmosphere at 60 °C for 10 h to activate the radical initiators. After cooled down, the solution mixture was spray-coated on one surface of Nafion^®^212 membrane defined as NR212. Prior to the coating, NR212 was immersed for 12 h in 75 wt % aqueous ethanol solution, and fixed in the silicon frame (dimension = 10 × 10 cm^2^) to keep the membrane flat during the spray coating process. The coated membrane was exposed at 60 °C for 1 h under UV irradiation with the dose of 1000 J cm^−2^ using a homemade UV chamber. Then, the membrane was treated in boiling 0.5 M HCl solution for 2 h and in boiling water for another 2 h to remove remaining unreacted monomers. The protonated membrane was immersed in 0.5 M NaCl solution at 30 °C for 2 days to convert it into the sodium (Na^+^) form membrane, and repeatedly washed in deionized water at 30 °C for 2 weeks to remove excess Na^+^ ions which do not participate in the formation of ionic bonding with sulfonic acid (–SO_3_^−^) groups and carboxylic acid (–COO^−^) groups in NR212 and synthetic AA-DVB layers, respectively [14]. The thickness of the AA-DVB layer in the double-layered membrane was controlled by varying the amount of the coated solution mixture. The resulting double-layered membrane was denoted as NR212/AA-DVB_C layer thickness. For example, NR212/AA-DVB_16 means the double-layered membrane composed of a NR212 layer and a AA-DVB layer with the thickness of 16 µm. AA-DVB membrane was prepared by casting on a glass plate (dimension = 10 × 10 cm^2^) instead of NR212 and by applying the same membrane formation procedures, and used as another reference.

### 2.3. Membrane Characterization

The Fourier-transform infrared (FT-IR) spectroscopy was carried out using the FT-IR spectrometer (Nicolet 6700 FTIR, Bruker, Billerica, MA, USA). The water uptake and water swelling behaviors of the resulting SWE membranes were observed by measuring their weight and volumetric differences between the dried and swollen membrane coupons. For this, each membrane coupon (dimension = 5 × 5 cm^2^) was dried under vacuum at 60 °C for one day, and fully hydrated in deionized water at 30 °C for one day. The ohmic resistance of the SWE membranes (*R*, membrane coupon size = 1 × 4 cm^2^) was measured in liquid water in the temperature range from 30 to 90 °C via four-point probe alternating current (a.c.) impedance spectroscopy using a VMP3 impedance spectrometer (Biologic, Seyssinet-Pariset, France) [15]. The Na^+^ ion conductivity (*σ*, S cm^−1^) was obtained using an equation of *σ* = *l*/(*R* × *S*). Here, *l* and *S* indicate the distance of counter and working electrodes and the cross-sectional area of the membrane coupon.

**Figure 2:**
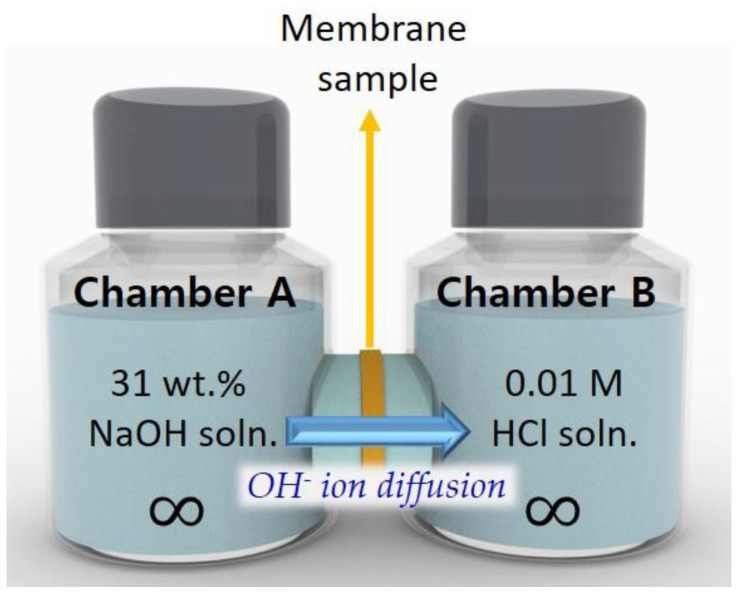
Schematic diagram of the two-chamber diffusion cell used in this study.

**Figure 3:**
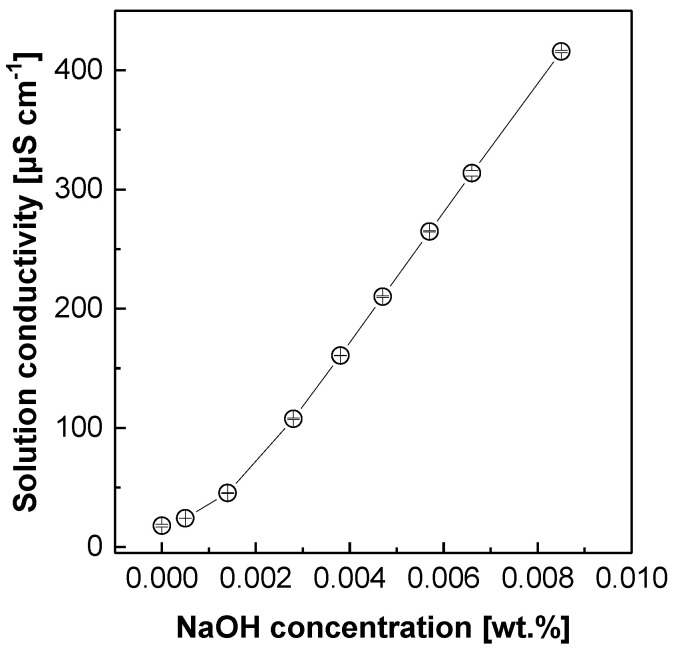
The correlation curve of NaOH solution conductivity with its concentration.

The concentration of OH^−^ ions transported through the SWE membranes [sample dimension (A) = 1 cm^2^] was measured at 90 °C as a function of time (*t*) using the two-chamber diffusion cell [16] in **Figure 2** where chamber A and B were filled with 31 wt % (~7.7 M, *C_A_*) NaOH solution and 0.01 M HCl solution of the same volume (i.e., 100 mL), respectively. A SWE membrane with a constant thickness (*L*) was placed between the two chambers. The OH^−^ ion concentration was obtained by monitoring the conductivity of the solution in chamber B with a potentiometric titrator (848 Titrino plus, Metrohm, Herisau, Swiss). Prior to the measurement, 9 kinds of NaOH solution with constant concentrations were made and their conductivity values were measured using the same titrator to obtain the standard curve in Figure 3. The OH^−^ ion permeability (*P_OH−_*, cm^3^ cm cm^−2^ sec^−1^) was determined using Equation (1) with the concentration gradient (*C_B_(t)*).

(1) $$P_{OH^{-}}\left[cm^{3}cm\;cm^{-2}sec^{-1}\right]=\frac{1}{A\;\left[cm^{2}\right]}\times \frac{C_{B}\left(t\right)}{C_{A}\left(t-t_{0}\right)\left[sec\right]}\times V_{B}\left[cm^{3}\right]\times L\left[cm\right]$$

The electrochemical SWE performance evaluation was conducted using a SWE cell (active area = 25 cm^2^, CNL energy, Seoul, Republic of Korea) at 90 °C. The platinum-coated titanium mesh electrodes (CNL energy, Republic of Korea) were used as both anode and cathode. The 31 wt % NaOH and 25 wt % NaCl were used as catholyte and anolyte, respectively. The pH of the anolyte was controlled to 2 via HCl titration to minimize the formation of active chloride species containing chlorate [2]. The current–voltage polarization curves were obtained using EDP 3050 power supplier (PNCYS, Uiwang, Korea), which was controlled by LabVIEW (National Instruments, Austin, TX, USA). The exact amount of H_2_ gas (molecular weight (MW_H2_) = 2.016 g mol^−1^) generated when a constant current (*I*) was applied for a constant time (*t*) was determined by measuring H_2_ concentration using a 490 Micro GC (Agilent Technologies, Santa Clara, CA, USA). The amount of NaOH (MW_NaOH_ = 39.997 g mol^−1^) was electrochemical measured via potentiometric titration using 848 Titrino plus titrator (Metrohm, Swiss), after H_2_ removal using a gas-liquid separator. The H_2_ and NaOH masses at time *t* (*m(t)*) and *0* (*m(0)*) were used to calculate hydrogen current efficiency (*η_H_*_2_) and caustic current efficiency (*η_NaOH_*, %) with Equation (2) [5,10,11]. Here, the *n* and *F* mean the mole number of electron accompanied by H_2_ and NaOH generation, and Faraday constant (96,485 C mol^−1^), respectively.

(2) ηH2 or NaOH[%]= (m(t)−m(0))[g](I [kA]×t[sec]n [mol]×F[C mol−1]×MWH2 or NaOH [g mol−1])×100

## 3. Results and Discussion

The C layers in SWE membranes should have high chemical resistance to harsh alkaline attacks (e.g., 31 wt % NaOH), particularly at 90 °C which is a commonly used SWE operation temperature. The selection of materials for the C layer is, however, very limited, since most polymeric materials are easily chemically decomposed under these tough conditions. One of feasible solutions is to select crosslinked polymeric networks where carboxylic acid groups exist. In the design of this polymeric network, AA was expected to form the polymer main chains containing carboxylic acid groups, while DVB was introduced as a crosslinker to induce the interconnected polymeric networks. BIE and AIBN were used to initiate the free radical polymerization via photolysis [17] and thermolysis [18] under the UV irradiation where heat is generated, respectively. The formation of crosslinked polymeric networks as shown in Figure 1 was confirmed on the basis of the solubility test of AA-DVB membrane conducted in representative polar aprotic solvents (i.e., N-methyl-2-pyrrolidone, dimethylacetamide, dimethylsulfoxide, dimethylformamide) at 60 °C for one day. The AA-DVB membrane was completely insoluble in all the polar aprotic solvents. It indicates that its polymer chains are successfully interconnected each other to make crosslinked polymeric networks. The carboxylic acid groups in the AA-DVB may also experience inter- and/or intramolecular dimerization [19,20] which induce physical crosslinking obtained as a result from hydrogen bonding between oxygen atoms in the carbonyl (C=O) groups and hydrogen atoms in the hydroxyl (–OH) groups as shown in Figure 1. The dimerization of the carboxylic acid groups is expected to be formed during the AA-DVB synthesis, and not to be easily broken even after 0.5 M NaCl treatment, because the interaction of carboxylic acid groups to alkali metal cations is much weaker than that of sulfonic acid groups, so carboxylic acid groups in the proton form (i.e., –COO^−^H^+^) would not be converted into those in the sodium form (i.e., –COO^−^Na^+^). Moreover, The AA-DVB membrane did not experience any weight change even when exposed to 31 wt % NaOH at 90 °C for one day. It means that the resulting AA-DVB layer is able to show high chemical resistance to the alkaline attack, which would be applied in the cathode side during a practical SWE operation, when used as the C layer in NR212/AA-DVB membranes.

**Figure 4:**
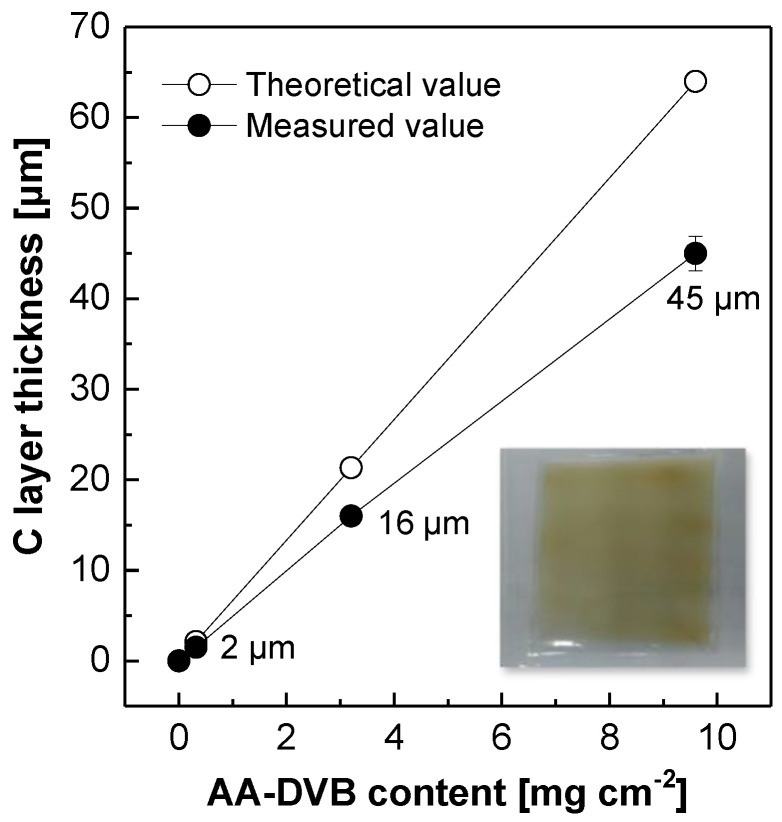
C layer thickness as a function of AA-DVB contents in double layered NR212/AA-DVB membranes.

Figure 4 shows how to control the C layer thickness in the double-layered NR212/AA-DVB membranes. The theoretical thickness of the C layer was calculated on the basis of the density of free-standing AA-DVB membrane in the dry state (see Table 1) under an assumption that a certain content of AA-DVB in the coating solution was used without any losses during the spray coating. On the other hand, the actual C layer thickness in the dry state was obtained by subtracting the NR212 thickness from the whole thickness values of NR212/AA-DVB membranes which was accomplished when the same AA-DVB contents were coated on the NR212 membrane. All the C layer thickness values linearly increase as a function of AA-DVB contents. There are, however, some gaps between the actual and theoretical thickness values. It may be related with the losses of AA-DVB coating solution during the spray coating. The targeted C layer thickness can be accomplished using these relationships.

In membrane appearance, NR212 used as S layer is transparent and colorless as shown in the edge of NR212/AA-DVB_2 membrane coupon in Figure 4. Meanwhile, AA-DVB layer on NR212 layer maintains its transparency, but show yellowish color. The yellowish color becomes darker as the C layer thickness gets thicker from 2 to 45 µm. The density values of NR212/AA-DVB membranes in Table 1 decrease as the thickness of AA-DVB layer increases, regardless of which humidity condition was applied for their density evaluation. The density trend is associated with a relatively low density of AA-DVB in comparison to NR212.

**Figure 5:**
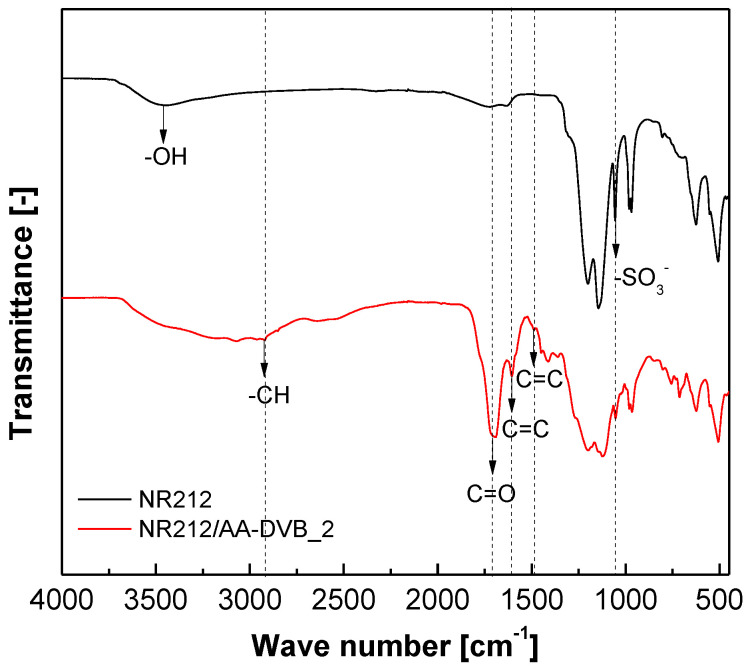
FT-IR spectra of NR212/AA-DVB_2 membrane compared with that of NR212.

Figure 5 shows FT-IR spectra of NR212/AA-DVB_2 to confirm the synthesis of the C layer on one surface of NR212. In the FT-IR spectra of NR212, a sharp peak associated with the asymmetric stretching vibration of –SO_3_– groups is observed at 1060 cm^−1^. After AA-DVB C layer formation, the peak is weakened owing to the limited penetration depth of FT-IR beam [21]. The NR212/AA-DVB_2 spectra has characteristic peaks assigned to C=C stretch in the aromatic rings of DVB, and –CH stretch in the main chain of AA-DVB layer [15,22]. NR212/AA-DVB_2 also exhibits a strong C=O vibration peak at around 1700 cm^−1^. This is attributable to carboxylic acid groups which come from AA monomers used during the C layer synthesis. Both NR212 and NR212/AA-DVB_2 membranes has a broad hydroxyl (–OH) peak at about 3500 cm^−1^ derived from absorbed water molecules. Note that it is difficult to completely remove water molecules tightly trapped within their matrices even in the dry state, owing to their hygroscopic property.

**Figure 6:**
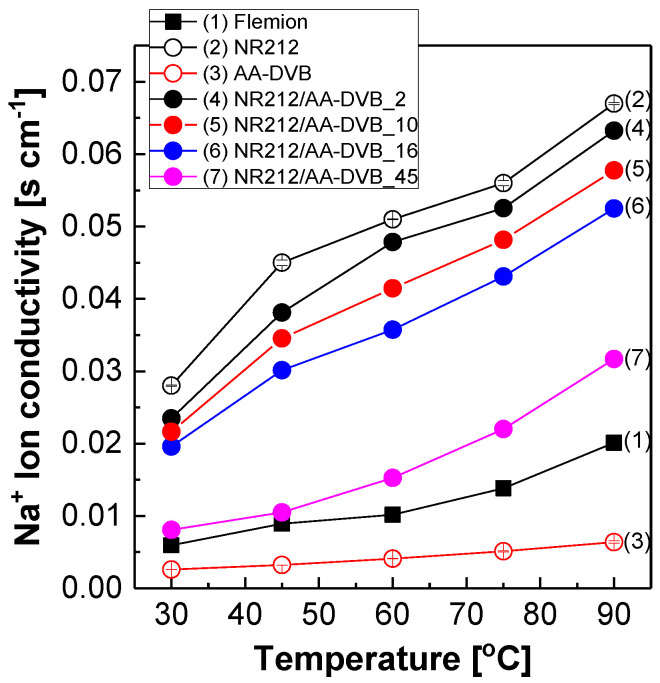
Na^+^ ion conductivity of NR212/AA-DVB membranes as a function of temperatures.

It is necessary to reveal the effect of the C layer on the transport behavior of Na^+^ ions through NR212/AA-DVB membranes for a desirable SWE membrane design. Figure 6 shows Na^+^ ion conductivity of NR212/AA-DVB membranes at the elevated temperatures. In all the membranes including Flemion^®^ and free-standing membranes (i.e., NR212 and AA-DVB) used as references, Na^+^ ion conduction becomes fast, as the temperature increases, owing to the enhanced diffusion of Na^+^ ions and the relatively fast molecular motion of their polymer chains. Among them, AA-DVB membrane exhibit the lowest Na^+^ ion conductivity. On the other hand, NR212 exhibits the highest Na^+^ conductivity, because of its well-defined hydrophilic-hydrophobic microphase-separated morphology derived from its side chains containing highly acidic sulfonic acid groups at their terminal ends and polytetrafluoroethylene main chains with extremely high hydrophobic character [23,24,25]. The Na^+^ conductivity values of AA-DVB membrane are at least 10 time lower than those of NR212 with the identical thickness of 50 µm. It may be due to the combined effect of the difference in acidity between carboxylic acid in AA-DVB and sulfonic acid groups in NR212, and restricted chain mobility associated with crosslinked AA-DVB matrix. The fairly reduced water uptake of AA-DVB in Table 1 also seems to negatively affect its Na^+^ ion conduction. Note that Na^+^ ions transport in the hydrated state via the formation of ionic complexes with water molecules. For example, the average hydration number of Na^+^ ion, which is defined as the number of water molecules strongly bound to a Na^+^ ion, is 4.6 in the temperature range from 47 to 95 °C [26]. Accordingly, the increase in C layer thickness causes the Na^+^ ion conduction through the double-layered membranes to be reduced. Even the reduced Na^+^ conductivity values of NR212/AA-DVB membranes are superior to those of Flemion^®^.

**Figure 7:**
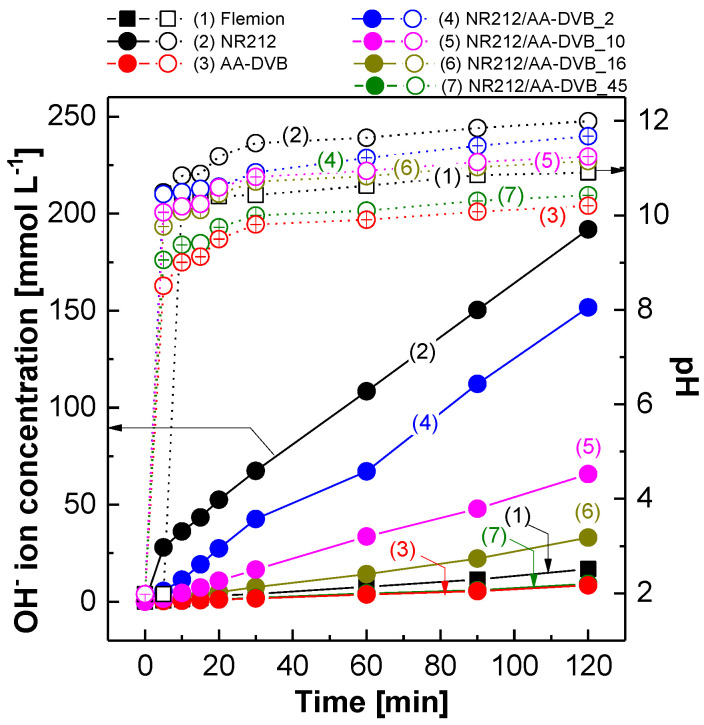
OH^−^ diffusion behavior of NR212/AA-DVB membranes over time.

Another important membrane parameter to determine electrochemical SWE characteristics is OH^−^ ion permeability, which is a measure of how fast OH^−^ ions can be diffused across the test membranes in the fully hydrated state where SWE operation is carried out. Figure 7 shows the changes of OH^−^ concentration and pH value in 0.01 M HCl solution filled in chamber B of the two-chamber diffusion cell as a function of times. Here the chamber B simulates the anolyte reservoir filled with the identical acidic solution in a SWE cell, and NaCl as the anodic reactant is excluded to electrochemically detect only OH^−^ ions permeated through the membranes. The initial pH value of the HCl solution is 2 at *t* = 0. When 31 wt % NaOH solution in the volume same to that of the HCl solution is introduced in the chamber A, OH^−^ ions begin to diffuse through the membranes. Consequently, the OH^−^ ion concentration in the acidic solution at the chamber B increases, irrespective of which membrane sample is used. The rate at which the OH^−^ concentration increases (i.e., concentration gradient of OH^−^ ions), becomes retarded, as the C layer thickness increases. It infers that the crosslinked C layer acts as an effective polymeric barrier to the diffusion of OH^−^ ions. Interestingly, the permeated OH^−^ ions rapidly raise the pH value of the initially acidic solution to the level higher than 10 within a short period of time. After 20 min, their pH values remain in the equilibrium state.

**Figure 8:**
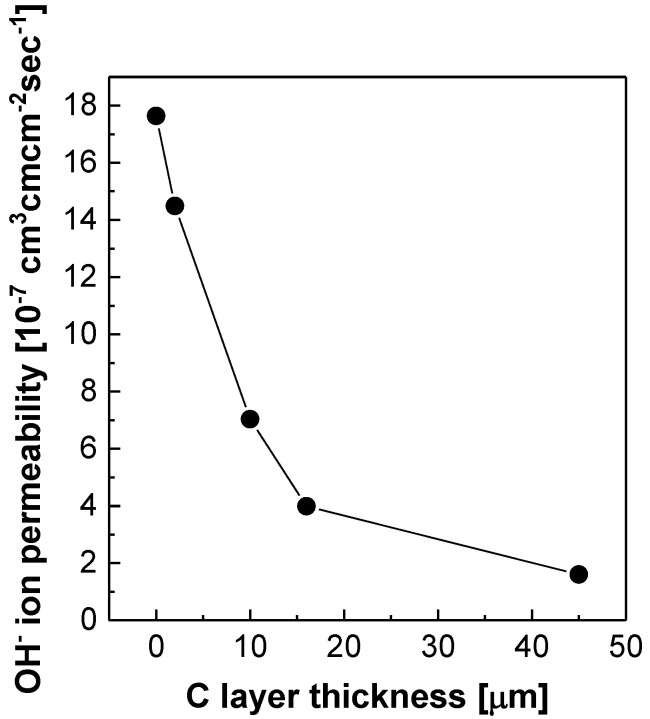
OH^−^ ion permeability of NR212/AA-DVB membranes as a function of C layer thickness.

Figure 8 displays the OH^−^ ion permeability obtained using the concentration gradient and the membrane dimension. The OH^−^ ion permeability is linearly reduced, until the C layer thickness reaches 16 µm. When a further increase in the C layer thickness is made, the reduction in the OH^−^ ion permeability is mitigated. Because of this tendency, NR212/AA-DVB_45 and free-standing AA-DVB have almost similar concentration gradient as shown in Figure 6. The OH^−^ permeability of NR212/AA-DVB_45 is not much different from that of free-standing AA-DVB membrane. It may mean that there are some limitations to strategies to reduce OH^−^ ion permeation by controlling only the thickness of the C layer in double layered SWE membranes.

**Figure 9:**
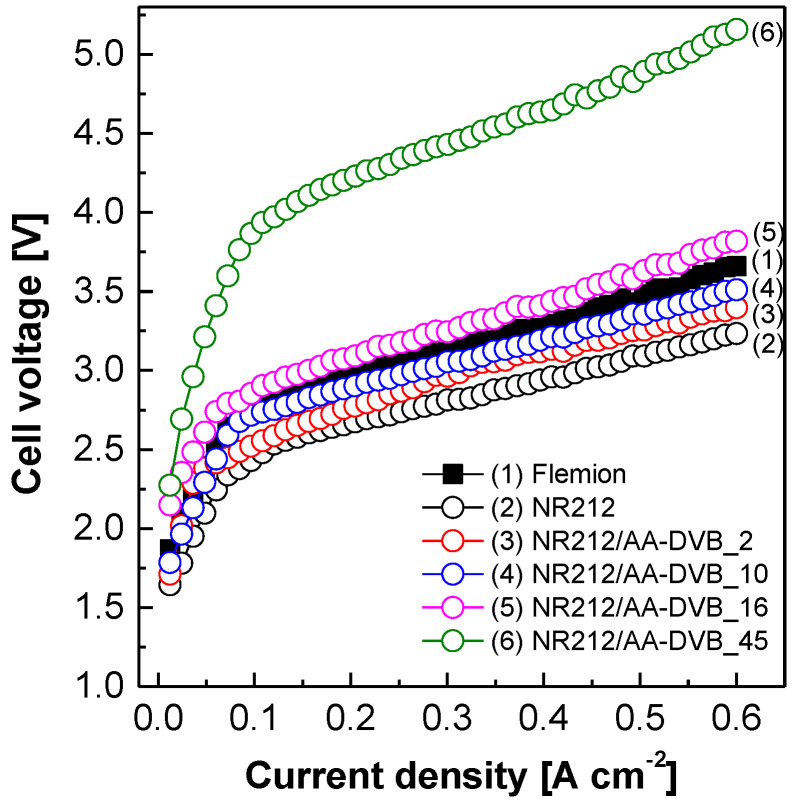
Current–voltage polarization behavior of NR212/AA-DVB membranes at 90 °C.

Figure 9 exhibits the effect of the C layer thickness on the electrochemical single cell performances of NR212/AA-DVB membranes obtained via chronoamperometry at 90 °C. The cell voltage values of all the membranes increase, as the applied current density values increase. The increment in the cell voltage of Flemion^®^ is much higher than that of NR212. The cell voltage of NR212 is 13.3% lower than that of Flemion^®^ at 0.6 A·cm^−2^, which is often chosen in industrial SWE applications. This difference may come from the Na^+^ conductivity of NR212 much higher than that of Flemion^®^ as shown in Figure 6. After the incorporation of the C layer on NR212, the cell voltage values of NR212/AA-DVB membranes at a constant current density rise when compared with that of NR212. Particularly, the slopes at the region of current density higher than 0.1 A cm^−1^ become faster, as the C layer thickness becomes thicker in the resulting double-layered membranes. It implies that the C layer also acts as the polymeric barrier to Na^+^ ion transport, lowering the electrochemical SWE performances of the corresponding membranes, since their slopes in the ohmic polarization region are proportional to their ohmic overpotentials accompanied by Na^+^ ion conduction across the membranes. The variation of electrochemical SWE performances according to the C layer thickness may also come from reduced transport rate of water molecules, maintaining ionic complexes with Na^+^ ions, through the thickened C layer, and lowered water flux [27]. The single cell performances of NR212/AA-DVB_2 and NR212/AA-DVB_10 are still superior to that of Flemion^®^.

**Figure 10:**
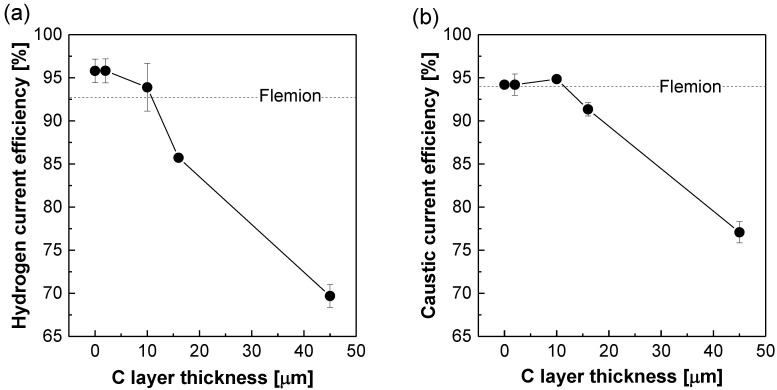
(**a**) Hydrogen and (**b**) caustic current efficiency of NR212/AA-DVB membranes as a function of C layer thickness at the constant current density of 0.6 A·cm^−2^. Here, the saline water electrolysis (SWE) operation temperature is 90 °C.

The electrochemical efficiency is a barometer to describe how the current is efficiently used for H_2_ and NaOH generation. The comparison of the electrochemical efficiency in the terms of Faraday efficiency can be very helpful in finding out the optimum thickness of C layer which plays a key role in blocking both Na^+^ and OH^−^ ions as polymeric barriers. Figure 10 shows the changes of hydrogen and caustic current efficiency at the constant current density according to the C layer thickness in NR212/AA-DVB membranes. Their hydrogen and caustic efficiency trends are mostly similar, but somewhat different in NR212/AA-DVB membranes with the C layer thinner than 10 µm. In the thickness range, the hydrogen current efficiency in Figure 10a is reduced as a result from lowered Na^+^ conduction. Meanwhile, the caustic current efficiency in Figure 10b is enhanced. It may be attributed to the reaction kinetics, particularly Le Chatelier’s principle [28], associated with unfavorable diffusion of OH^−^ ions from cathode reservoir to anode reservoir in the SWE cell. That is, H_2_ and NaOH are simultaneously generated during the SWE reaction (e.g., the related half reaction: 2H_2_O + 2e^−^ → H_2_ + 2OH^−^). When OH^−^ ions are diffused to the anode reservoir across the membranes, the NaOH production is relatively reduced on the basis of the theoretical stoichiometric ratio of NaOH to H_2_. This peculiar trend can be alleviated by increasing the thickness of the C layer up to 10 µm. In contrast, NR212/AA-DVB membranes with the thickness higher than 10 µm experience the reduction of their caustic current efficiency owing to the conflicting effect of Na^+^ ion transport.

**Figure 11:**
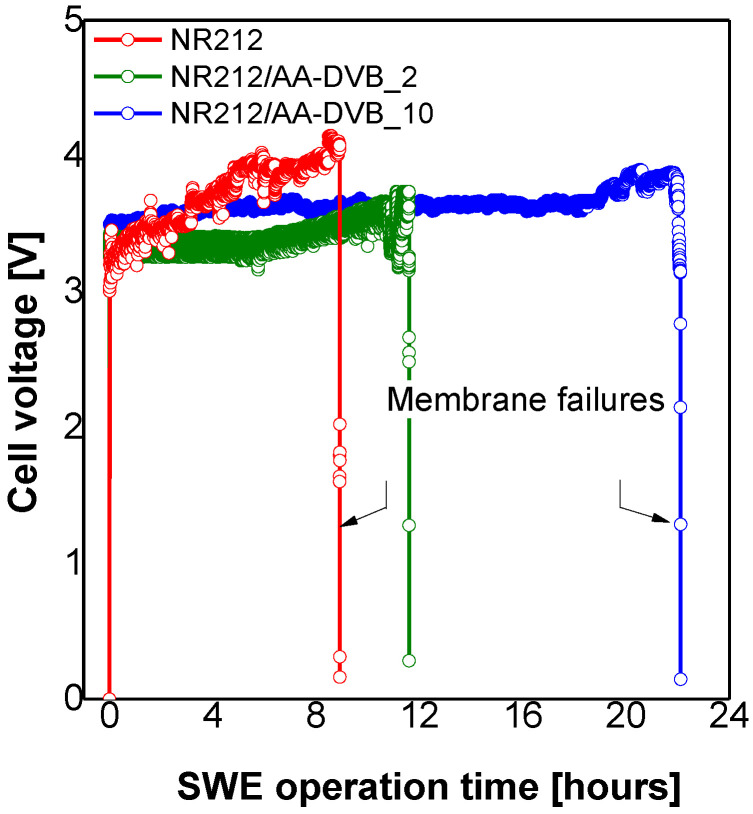
SWE durability test results of NR212/AA-DVB membranes obtained at the constant current density of 0.6 A·cm^−2^. Here, the SWE operation temperature is 90 °C.

In addition to electrochemical single cell performance and efficiency, the other barometer needed for a desirable SWE membrane design is the electrochemical durability under practical SWE operation conditions. Figure 11 shows the changes of electrochemical single cell performances obtained when the constant current density of 0.6 A·cm^−2^ is continuously applied. In NR212 used for comparison, the cell voltage increases rapidly up to with high fluctuations within 2.5 h. After 9 h elapse, the cell voltage largely deviates from the nominal value of ~3.2 V, and suddenly falls below the theoretical SWE cell voltage of 2.2 V. This pattern is associated with membrane failures including the formation of pinholes and defects, and membrane thickness reduction. After the incorporation of C layer, the rate at which the overpotential increases becomes slow, and the degree of the voltage fluctuation is also lessened. The C layer accounting for 17% of the total thickness in case of NR212/AA-DVB_10 contributes to improving the membrane lifetime by 2.5 time in comparison with that of NR212 composed of a single PFSA matrix. When comprehensively considering the SWE membrane characteristics, the optimum C layer thickness for the desirable double-layered membrane formation is 10 µm.

## 4. Conclusions

Double-layered membranes in the form of CEMs were successfully prepared with design strategies composed of the incorporation of crosslinked C layer containing carboxylic acid groups via the free radical polymerization under UV irradiation onto one surface of a commercially available perfluorinated NR212 membrane used for S layer containing sulfonic acid groups, and the thickness control of the C layer. After the addition of the C layer, the resulting NR212/AA-DVB membranes exhibited reduced Na^+^ ion conductivity and improved barrier property to OH^−^ ions, when compared with those of NR212. These trends were more significantly observed as the C layer thickness increases. The electrochemical SWE single cell performances of the resulting double-layered membranes were more affected by their Na^+^ ion conductivity rather than OH^−^ ion permeability. The double-layer membranes with thin C layer in the thickness range of 10 µm showed single cell performances higher than the state-of-the art SWE membrane, Flemion^®^. The hydrogen current efficiency was more dependent on Na^+^ ion conduction. On the other hand, the caustic current efficiency was influenced by both OH^−^ ion permeation and Na^+^ ion conduction. The OH^−^ ion diffusion through the SWE membranes lowering the NaOH production, was alleviated by increasing the C layer thickness up to 10 µm. This thickness control was helpful in improving caustic current efficiency. The further increase in the C layer thickness retarded Na^+^ conduction, and led to the highly reduced caustic current efficiency. The addition of the thin C layer contributed to extended service life of the corresponding SWE membranes. It has been proven that the proposed design strategies is very useful for preparing effective SWE membranes with high single cell performances and electrochemical efficiency values. Even the extended lifetimes of the double-layered membranes are, however, insufficient to be used as alternatives to Flemion^®^ for a long-term SWE operation (e.g., at least 1–2 years). Our on-going studies are focused on the approaches to effectively incorporate a chemically robust C layer made up of PFCA ionomers on the S layer with much enhanced Na^+^ ion conductivity (e.g., S layer made using PFSA ionomers with low equivalent weight values). We have also attempted to monitor the effect of free volume in the C layers with the identical chemical architectures on SWE membrane characteristics.

## Figures and Tables

**Table 1:** Fundamental Characteristics of NR212/AA-DVB Membranes.

Sample	Thickness *^a^*	Dry Density	Wet Density	Water Uptake	Dimensional Change [%]		
	[µm]	[g cm^−3^]	[g cm^−3^]	[%]	Width	Length	Thickness
NR212	50	1.98	1.75	22.4	11.1	11.1	12.6
AA-DVB	50	1.50	1.46	3.7	1.1	1.1	4.8
NR212/AA-DVB_2	52	1.87	1.72	14.0	6.4	6.4	9.2
NR212/AA-DVB_10	60	1.76	1.69	13.6	4.2	4.2	8.9
NR212/AA-DVB_16	66	1.75	1.68	13.4	4.2	4.2	8.6
NR212/AA-DVB_45	95	1.67	1.63	6.8	2.1	2.1	5.3
Flemion^®^	220	1.26	1.20	17.5	6.4	6.4	8.4

*^a^* Measured in the dry state.

## References

[1] Du F., Warsinger D.M., Urmi T.I., Thiel G.P., Kumar A., Lienhard J.H.V. (2018). Sodium hydroxide production from seawater desalination brine: Process design and energy efficiency. Environ. Sci. Technol..

[2] Karlsson R.K.B., Cornell A. (2016). Selectivity between oxygen and chlorine evolution in the chlor-alkali and chlorate processes. Chem. Rev..

[3] Shiroki H., Hiyoshi T., Ohta T. (1992). Recent development and operation dynamics of new ion exchange membrane series aciplex^®^-F from asahi chemical. Modern Chlor-Alkali Technology.

[4] Lee J.H., Lee J.H., Park I.K., Lee C.H. (2018). Techno-economic and environmental evaluation of CO_2_ mineralization technology based on bench-scale experiments. J. CO2 Util..

[5] Lee J.H., Park I.K., Duchesne D., Chen L., Lee C.H., Lee J.H. (2020). Saline water electrolysis system with double-layered cation exchange membrane for low-energy consumption and its application for CO_2_ mineralization. J. CO2 Util..

[6] Echardt J., Kornmueller A. (2009). The advanced EctoSys electrolysis as an integral part of a ballast water treatment system. Water Sci. Technol..

[7] Lacasa E., Tsolaki E., Sbokou Z., Rodrigo M.A., Mantzavinos D., Diamadopoulos E. (2013). Electrochemical disinfection of simulated ballast water on conductive diamond electrodes. Chem. Eng. J..

[8] Chlistunoff J. (2005). Advanced Chlor-Alkali Technology: Final Technical Report LAUR 05-2444.

[9] De Bruijn F.A., Makkus R.C., Mallant R.K.A.M., Janssen G.J.M. (2007). Chapter five—Materials for state-of-the-art PEM fuel cells, and their suitability for operation above 100 °C. Adv. Fuel Cells.

[10] Park I.K., Ahn C.Y., Lee J.H., Lee D.W., Lee C.H., Cho Y.H., Sung Y.E. (2019). Three-dimensionally interconnected titanium foam anode for an energy-efficient zero gap-type chlor-alkali electrolyzer. Int. J. Hydrogen Energy.

[11] Park I.K., Cha W.J., Lee C.H. (2020). Saline water electrolysis membranes prepared via the simultaneous irradiation of electron-beam. ECS Trans..

[12] Elliott J.E., Macdonald M., Nie J., Bowman C.N. (2004). Structure and swelling of poly(acrylic acid) hydrogels: Effect of pH, ionic strength, and dilution on the crosslinked polymer structure. Polymer.

[13] Huang J., Wan S., Guo M., Yan H. (2006). Preparation of narrow or mono-disperse crosslinked poly((meth)acrylic acid)/iron oxide magnetic microspheres. J. Mater. Chem..

[14] Xie W., Geise G.M., Freeman B.D., Lee C.H., McGrath J. (2012). Influence of processing history on water and salt transport properties of disulfonated polysulfone random copolymers. Polymer.

[15] Fu Y.W., Sun W.F., Wang X. (2020). UV-initiated crosslinking reaction mechanism and electrical breakdown performance of crosslinked polyethylene. Polymers.

[16] Kim D.S., Park H., Rhim J.W., Lee Y.M. (2004). Preparation and characterization of crosslinked PVA/SiO_2_ hybrid membranes containing sulfonic acid groups for direct methanol fuel cell applications. J. Membr. Sci..

[17] Lipscomb N.T., Tarshiani Y. (1988). Kinetics and mechanism of the benzoin isobutyl ether photoinitiated polymerization of styrene. J. Polym. Sci. Part A.

[18] Bevington J.C. (1987). Initiation of polymerization: Azo compounds and peroxides. Makromol. Chem. Macromol. Symp..

[19] Chen J., Brooks C.L., Scheraga H.A. (2008). Revisiting the carboxylic acid dimers in aqueous solution: Interplay of hydrogen bonding, hydrophobic interactions, and entropy. J. Phys. Chem. B.

[20] Tsivintzelis I., Kontogeorgis G.M., Panayiotou C. (2017). Dimerization of carboxylic acids: An equation of state approach. J. Phys. Chem. B.

[21] Tang C., Kwon Y.N., Leckie J. (2007). Probing the nano- and micro-scales of reverse osmosis membranes—A comprehensive characterization of physiochemical properties of uncoated and coated membranes by XPS, TEM, ATR-FTIR, and streaming potential measurements. J. Membr. Sci..

[22] Zhao X.D., Zhao H., Sun W.F. (2020). Significantly improved electrical properties of crosslinked polyethylene modified by UV-initiated grafting MAH. Polymers.

[23] Kreuer K.D. (1996). Proton conductivity: Materials and applications. Chem. Mater..

[24] Kreuer K.D., Paddison S.J., Spohr E., Schuster M. (2004). Transport in proton conductors for fuel-cell applications: Simulations, elementary reactions, and phenomenology. Chem. Rev..

[25] Park C.H., Lee C.H., Guiver M.D., Lee Y.M. (2011). Sulfonated hydrocarbon membranes for medium-temperature and low-humidity proton exchange membrane fuel cells (PEMFCs). Prog. Polym. Sci..

[26] Rempe S.B., Pratt L., Hummer G., Kress J.D., Martin R.L., Redondo A. (2000). The hydration number of Li^+^ in liquid water. J. Am. Chem. Soc..

[27] Mandal M., Huang G., Hassan N.U., Peng X., Gu T., Brooks-Starks A.H., Bahar B., Mustain W.E., Kohl P.A. (2019). The importance of water transport in high conductivity and high-power alkaline fuel cells. J. Electrochem. Soc..

[28] Russell J.M. (1988). Simple models for teaching equilibrium and Le Châtelier’s principle. J. Chem. Educ..

